# Supplementary material for: Conditional chemoconnectomics (cCCTomics) as a strategy for efficient and conditional targeting of chemical transmission
Source: eLife. 2024 Apr 30;12:RP91927. doi: 10.7554/eLife.91927 (PMC11060718; doi:10.7554/eLife.91927)
Supplement: Figure 3—source data 3. [file elife-91927-fig3-data3.zip › Figure 3 source data3 part 2.zip!/Figure 3-Source data 3-part2/1052-13-CRISPResso_Report_ejedac2.zip!/CRISPResso_on_1052_15/9.Alleles_frequency_table_around_sgRNA_agtggacaaactggtacatg.pdf]

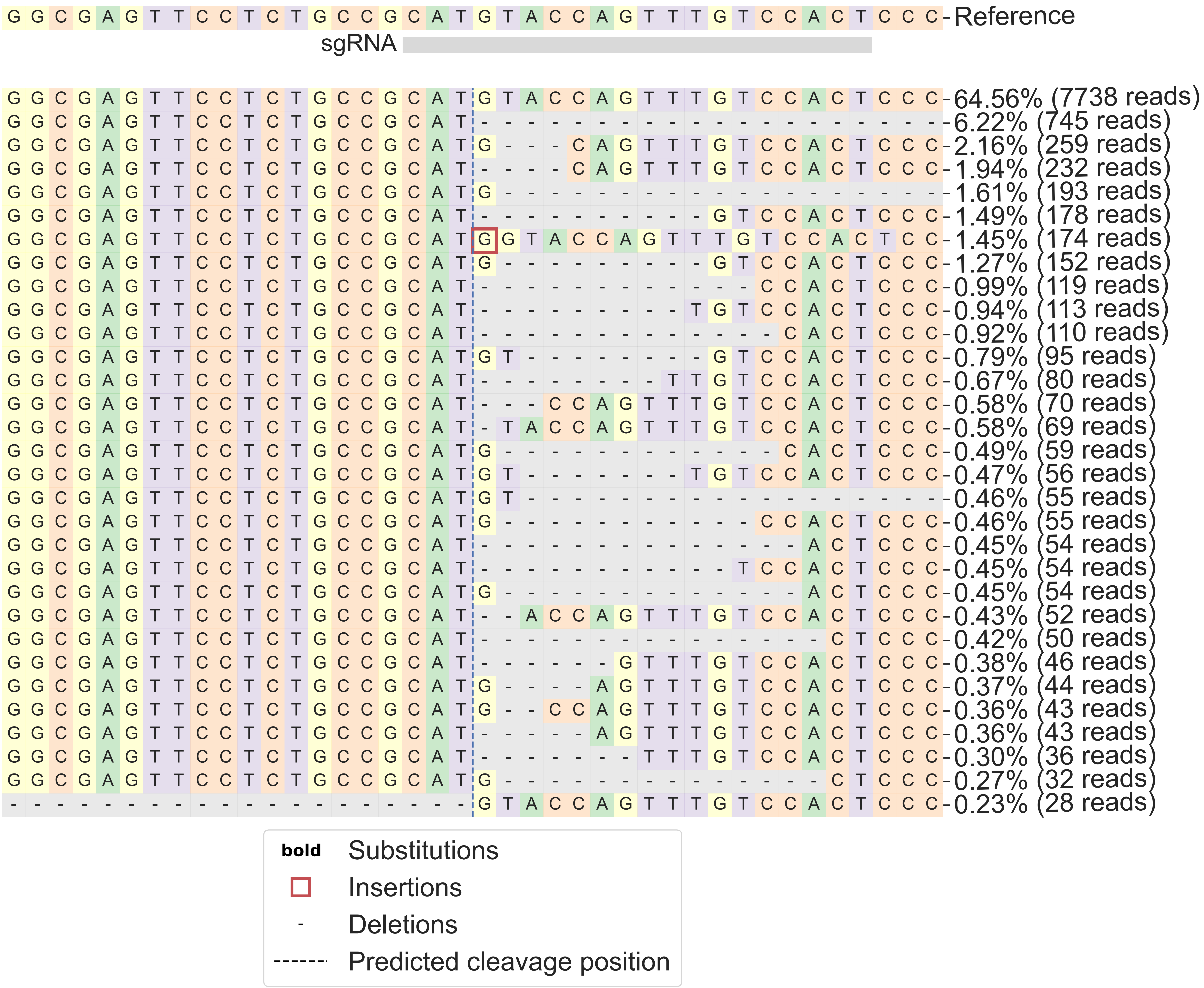

Reference
sgRNA
64.56% (7738 reads)
6.22% (745 reads)
2.16% (259 reads)
1.94% (232 reads)
1.61% (193 reads)
1.49% (178 reads)
1.45% (174 reads)
1.27% (152 reads)
0.99% (119 reads)
0.94% (113 reads)
0.92% (110 reads)
0.79% (95 reads)
0.67% (80 reads)
0.58% (70 reads)
0.58% (69 reads)
0.49% (59 reads)
0.47% (56 reads)
0.46% (55 reads)
0.46% (55 reads)
0.45% (54 reads)
0.45% (54 reads)
0.45% (54 reads)
0.43% (52 reads)
0.42% (50 reads)
0.38% (46 reads)
0.37% (44 reads)
0.36% (43 reads)
0.36% (43 reads)
0.30% (36 reads)
0.27% (32 reads)
0.23% (28 reads)
bold Substitutions
Insertions
Deletions
Predicted cleavage position
